# Combined Effects from Dual Incorporation of ATBC as Plasticizer and Mesoporous MCM-41 as Nucleating Agent on the PLA Isothermal Crystallization in Environmentally-Friendly Ternary Composite Systems

**DOI:** 10.3390/polym15030624

**Published:** 2023-01-25

**Authors:** Enrique Blázquez-Blázquez, Rosa Barranco-García, Tamara M. Díez-Rodríguez, María L. Cerrada, Ernesto Pérez

**Affiliations:** Instituto de Ciencia y Tecnología de Polímeros (ICTP-CSIC), Juan de la Cierva 3, 28006 Madrid, Spain

**Keywords:** PLA, ATBC, MCM-41, plasticizer, nucleating agent, crystallization rate, glass transition, crystalline polymorphs

## Abstract

Different materials, based on an *L*-rich polylactide (PLA) as matrix, acetyl tri-*n*-butyl citrate (ATBC) as plasticizer, and mesoporous Mobile Crystalline Material.41 (MCM-41) particles as nucleating agent, were attained by melt extrusion. These materials are constituted by (a) binary blends of PLA and ATBC with different contents of the latest; (b) a dual compound of PLA and a given amount of MCM-41 silica (5 wt.%); and (c) ternary composites that include PLA, ATBC at several compositions and mesoporous MCM-41 at 5 wt.%. Influence of the incorporation of the plasticizer and nucleating particles has been comprehensively analyzed on the different phase transitions: glass transition, cold crystallization, melt crystallization and melting processes. Presence of both additives moves down the temperature at which PLA phase transitions take place, while allowing the PLA crystallization from the melt at 10 °C/min in the composites. This tridimensional ordering is not noticeable in the pristine PLA matrix and, accordingly, PLA crystallization rate is considerably increased under dynamic conditions and also after isothermal crystallization from either the melt or the glassy state. An important synergistic effect of dual action of ATBC and MCM-41 has been, therefore, found.

## 1. Introduction

Poly(lactide) (PLA), a biodegradable aliphatic polyester with an excellent profile in properties, could play a major role in future polymers markets for numerous applications because its monomer comes from renewable resources. PLA shows, however, some shortcomings, such as its brittleness or its relatively reduced crystallization ability under common processing conditions [1]. The former is ascribed to location of its glass transition temperature (T_g_), which appears at around 60 °C, and the latest is associated with its low crystallization rate. The position of T_g_ can be easily adjusted by adding plasticizers, while its three-dimensional ordering capacity can be modulated either by incorporating nucleating agents, since they contribute to lowering the activation energy of nucleation, or by adding plasticizers, due to the increase in the overall mobility of polymeric chains that their presence plays. Nevertheless, the joint incorporation of these additives, plasticizer and nucleanting agent, is an approach little explored in literature [2], although it can be quite attractive from academic and practical standpoints. On one hand, presence of the former can reduce the significant and negative impact that the physical aging, commonly observed in PLA, exerts in its behavior, which promotes a deficient dimensional stability and changes in other key parameters. On the other hand, addition of a nucleant agent will boost crystallization capability of PLA and its rate, inducing the enhancement of some important properties, such as overall strength, thermal resistance or gas barrier. Accordingly, a favorable dual action of both would lead to a balanced and improved response in either amorphous or crystalline phases within the PLA matrix and, subsequently, in the ultimate performance of the resultant ternary materials.

Plasticizers are widely used to enhance flexibility, processability and ductility of polymers. Nevertheless, different variables must be taken into consideration for achieving an efficient effect, which include the nature of matrix, the type and optimal percentage of plasticizer, its thermal stability at the processing temperature, among others. In semicrystalline polymers, such as PLA, incorporation of a plasticizer should reduce the T_g_ and, additionally, allow the shift to lower temperatures of the crystallization window. The plasticizer efficiency is also noticeable through a melting point depression of the corresponding crystalline phase [3,4]. Numerous types of plasticizers of low molecular weight have been studied for PLA, such as the bishydroxymethyl malonate (DBM) [5], the glucose monoesters and partial fatty acid esters, the citrates [6], among others. Nonetheless, they show often the problem of migration, due to their high mobility within the PLA macrochains. Thus, plasticizers with higher molecular weight and, accordingly, lower mobility have been also evaluated, such as the poly(ethylene glycol) (PEG) [6,7,8], the poly(propylene glycol) (PPG) [9], the atactic poly(3-hydroxybutyrate) (a-PHB) [10,11] and the oligoesteramide (DBM-oligoesteramide) [5,12,13]. Furthermore, the plasticizer selection for PLA is also restricted by the legislative or technical requirements of the final applications if these are, for instance, as food packaging or in the biomedical field. Therefore, the choice becomes even more complex [5,13]. The good solubility of PLA in the citrate plasticizers, owing to the polar interactions between the ester groups of PLA and the plasticizer [14], makes the acetyl tri-*n*-butyl citrate (ATBC), which is also biodegradable and non-toxic compound, a successful and common alternative to be added to PLA [1,4,13].

Global rates of the nucleation and the crystallization in the PLA under homogeneous conditions are, as aforementioned, relatively low. Accordingly, the addition of nucleating agents is a strategy to promote both processes. These additives allow increasing the number of primary nucleation sites and reducing the nucleation induction period. Different physical agents have been analyzed in PLA for this role, including organic, mineral and mineral-organic hybrids [15]. Talc has been commonly considered as a reference in PLA to evaluate the nucleation capacity of other agents [15,16]. Carbon nanotubes, either neat or modified, have been also employed [15] due to their high aspect ratio and excellent mechanical, thermal and electrical properties. Recently, the mesoporous silica has been used as well. In particular, the SBA-15 particles have been proved to play an important role in the PLA crystallization in composites prepared either by melt extrusion [17,18] or by solution mixing [19]. Mesoporous SBA-15 is constituted by ordered hexagonal arrangements within the particles, which are developed during the synthesis and consist in hollow channels with diameters from around 7 to 10 nm [20].

The aim of this research is to evaluate the joint influence of the dual incorporation of a plasticizer and a mesoporous silica in the crystallization process of PLA. Then, ATBC has been chosen for the former and MCM-41 as silica in the preparation of ternary systems. This plasticizer is selected because the good results, reported in literature, that its incorporation involves. The MCM-41 mesoporous silica has been chosen since it shows similar morphological characteristics [21] to those found in SBA-15, although its hexagonal arrangement displays lower pore size due to the template required for its preparation. Furthermore, its synthesis is also less expensive than that for the SBA-15 manufacture. Binary materials constituted, on one hand, by PLA and different contents of ATBC and, on the other hand, by PLA and a given amount of MCM-41 have been also prepared additionally to the ternary PLA/plasticizer/MCM-41 composites, in order to study the existence or lack of a synergistic effect of the two additives. A thorough analysis has been performed by differential scanning calorimetry (DSC) under isothermal conditions, from either the molten or the glassy states, at different temperatures to fully understand the role that both additives exerts on PLA crystallization.

## 2. Materials and Methods

A commercially available polylactide (PLA) from NatureWorks^®^ (labeled as Ingeo™ Biopolymer 6202D, with a density of 1.24 g/cm^3^, and a content in *L*-isomer units of about 98 mol%) is used in this study. Its weight-average molecular weight (M_w_) and molecular weight dispersity are 118,600 g/mol and 1.6, respectively, as determined from gel permeation chromatography (GPC) [22].

**Scheme 1:**
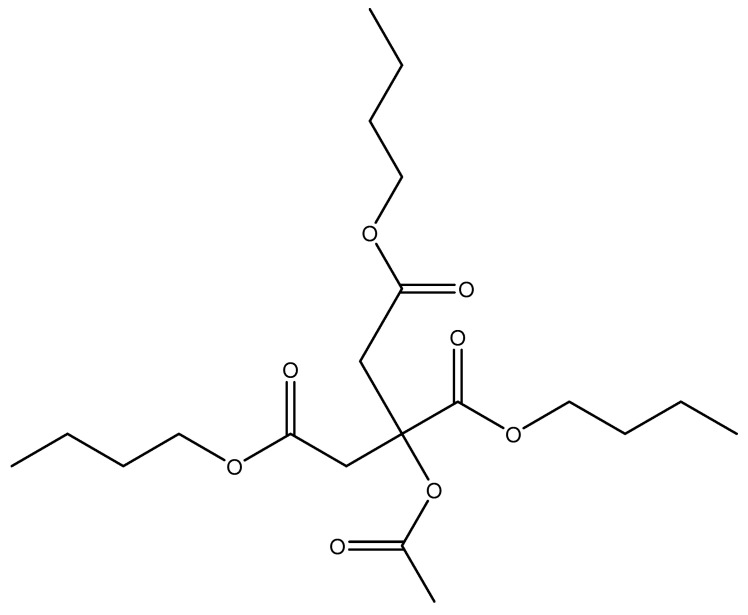
Chemical structure of the ATBC plasticizer.

The ATBC (CAS 77-90-7) plasticizer was purchased from Sigma–Aldrich (Madrid, Spain) with purity equal or greater than 98.0%. Its chemical structure is shown in Scheme 1. It has a molecular weight of 402.5 g/mol and a boiling temperature of 331 °C.

The MCM-41 particles were purchased from Sigma-Aldrich (specific surface area, S_BET_ = 966 m^2^/g; average mesopore diameter, D_p_ = 2.9 nm [23]) and were used as received.

PLA, PLA-ATBC binary blends at different plasticizer contents, binary composite of PLA and MCM-41, along with PLA/ATBC/MCM-41 ternary composites were prepared through melt processing in a micro-conical twin screw Thermo Scientific Haake Minilab miniextruder operating at 190 °C, with a screw speed of 100 rpm, using a mixing time of 4 min after the full feeding. Sample labeling and nominal composition are detailed in Table 1. PLA and MCM-41 were dried previous extrusion. The former was initially placed in an oven at 85 °C for 20 min followed by a drying under vacuum at 85 °C for 2 h. The MCM-41 silica was dried under vacuum at 100 °C for 24 h.

All of these materials were subsequently processed by compression molding in a hot-plate Collin press. Initially, the material was maintained at a temperature of 190 °C without pressure for 2 min and, then, a pressure of 25 bar was applied for 5 min. Afterward, a cooling process at the relatively rapid rate of around 80 °C/min and at a pressure of 25 bar was applied to the different composites from their molten state to room temperature. These original compression-molded films were totally amorphous, as shown below from the zero neat enthalpy involved in the first melting DSC curves.

Morphological details of the mesoporous MCM-41 were obtained by transmission electron microscopy (TEM). Measurements were performed at room temperature in a 200 kV JEM-2100 JEOL microscope. The particles were dispersed in acetone in an ultrasonic bath for 5 min and then deposited in a holder prior to observation.

Fracture surface in different sections of the films was evaluated by scanning electron microscopy (SEM) using a Philips XL30 microscope The samples were coated with a layer of 80:20 Au/Pd alloy and deposited in a holder before visualization.

Thermogravimetric analysis (TGA) was performed in a Q500 equipment of TA Instruments, under air atmosphere at a heating rate of 10 °C/min. Maximum degradation temperatures of the distinct materials are determined, as well as the actual amount of the two additives incorporated into the extruded materials prepared by extrusion.

Wide angle X-ray Diffraction (WAXD) patterns were recorded at room temperature in the reflection mode, by using a Bruker D8 Advance diffractometer provided with a PSD Vantec detector. Cu Kα radiation (λ = 0.15418 nm) was used, operating at 40 kV and 40 mA. The parallel beam optics was adjusted by a parabolic Göbel mirror with horizontal grazing incidence Soller slit of 0.12° and LiF monochromator. The equipment was calibrated with different standards. A step scanning mode was employed for the detector. The diffraction scans were collected with a 2θ step of 0.024° and 0.2 s per step.

Calorimetric analyses were carried out in a TA Instruments Q100 calorimeter connected to a cooling system and calibrated with different standards. The sample weights were around 3 mg. A temperature interval from −30 to 190 °C was studied at a heating rate of 10 °C/min in dynamic experiments. Isothermal measurements have been also performed either from the molten or the glassy state.

Details for isothermal experiments from the melt are the following: a rapid cooling, at 60 °C/min, was applied from 190 °C to the desired crystallization temperature, T_c_, which was isothermally maintained for the required time. Subsequently, the heating curve at 10 °C/min is registered. The crystallization temperatures where extended to those values where the crystallization is complete after a reasonable time (total crystallization times requiring longer than around 100 min show already a very bad signal-to-noise ratio).

Details for isothermal experiments from the glassy state are the next: starting again at 190 °C, a rapid cooling is carried out at 60 °C/min, now until below the glass transition of the sample. Then, the desired crystallization temperature is reached by heating also at 60 °C/min, being isothermally maintained for the required time. Afterward, the subsequent heating curve is registered at 10 °C/min. The crystallization temperatures where extended up to those temperatures before the cold crystallization is observed in the heating ramp for reaching the crystallization temperature.

All the DSC curves presented throughout this work are actual curves without normalization for the real content of PLA in the sample. That normalization has been performed, however, for the enthalpies used to estimate crystallinity degree of the different sample experiments, i.e., the values achieved are then the normalized crystallinity. For the determination of the crystallinity, 93.1 J/g was used as the enthalpy of fusion of a perfectly crystalline material [24,25].

## 3. Results and Discussion

**Figure 1:**
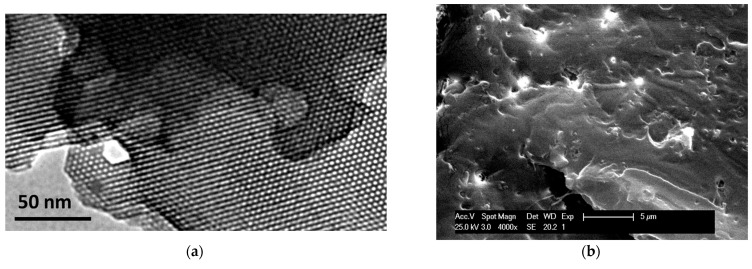
(**a**) TEM micrographs of MCM-41. (**b**) SEM fracture picture for the PLAM5 composite.

Figure 1a shows the ordered hexagonal arrangements that constitute the interior of the particles of the mesoporous MCM-41 silica. This inner part consists in a well-defined hollow channel structure with pore diameters of around 3 nm, where PLA chain segments and/or molecules of plasticizer can be embedded. Experimental conditions used during extrusion turn out appropriate, as deduced from the SEM fracture picture found in the PLAM5 composite depicted in Figure 1b, where a homogeneous distribution of silica within the matrix of PLA is exhibited together with the absence of inorganic domains of large size.

**Figure 2:**
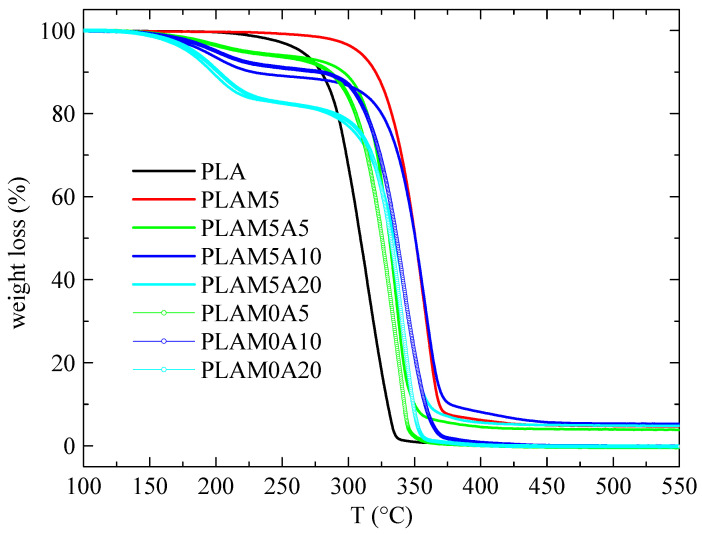
TGA curves under air atmosphere for the different materials analyzed.

Thermogravimetry measurements are carried out in order to learn how presence of: (a) individual plasticizer in the PLA-ATBC blends, (b) MCM-41 in binary composites or (c) both additives in plasticized and filled PLA ternary materials, affects the thermal stability of this polymeric matrix. Furthermore, these TGA experiments allow estimating the actual material content for the different constituents (PLA, ATBC plasticizer and MCM-41 silica) in the several composites. Figure 2 shows the TGA curves under air atmosphere for the different samples, indicating that PLA maximum degradation temperature is shifted to higher temperatures (see Table 2) by incorporation of ATBC plasticizer in the binary blends.

At the lowest amount of ATBC, the increase is around 23 °C. This rise is even slightly larger for the two higher contents of ATBC used. A much important displacement is ob-served in the PLAM5 specimen, which is the binary composite of PLA and MCM-41 silica. Thus, inclusion of mesoporous MCM-41 into PLA by extrusion leads to a very remarkable improvement of PLA thermal stability. The shift of the maximum degradation temperature (T_max_^PLA^) to higher temperatures has been also previously described when adding talc to PLA [26] and in PLA composites incorporating modified clays [27]. The combined effect of ATBC plasticizer and MCM-41 silica leads to an increase of the PLA thermal stability compared with that exhibited by the neat polymeric matrix, showing an optimized T_max_^PLA^ value in the sample PLAM5A10. This type of behavior of a maximum effect at intermediate plasticizer content has been already reported [4]. Nevertheless, a straightforward variation with the amount of the two additives is not noticed. Thus, the values found in PLAM5A5 and PLAM5A20 are analogous to those observed in PLAM0A5 and PLAM0A20, respectively, while that shown by PLAM5A10 is similar to that noted in PLAM5. A full understanding of the thermal stability of these ternary systems requires a more complete analysis, which is out of the scope of this investigation.

Table 2 also displays that the T_1%_ in air is considerably smaller in the materials that contain the ATBC additive, since this plasticizer is the first compound that is lost through evaporation. Moreover, the actual content of the different constituents in the several materials are also collected in Table 2.

**Figure 3:**
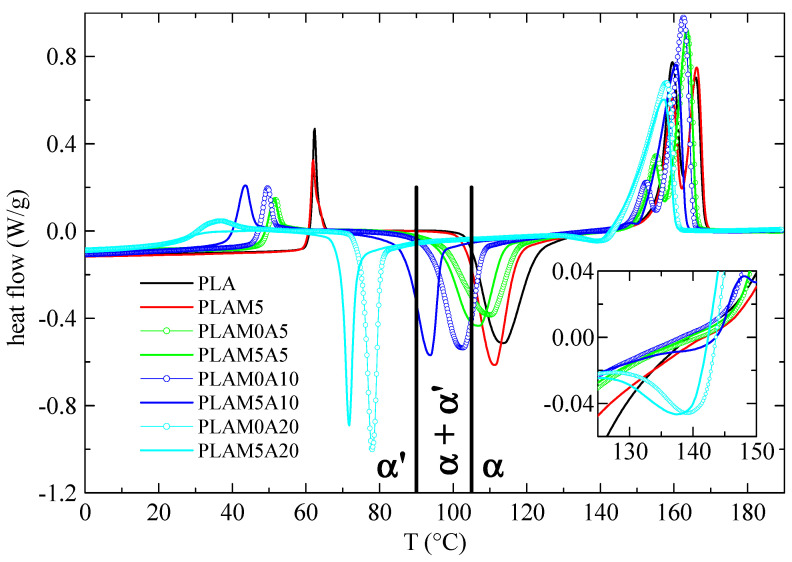
DSC first melting curves (endo up) at 10 °C/min of the different compression-molded materials. The vertical lines are the corresponding values reported in reference [22], showing the temperature intervals where the pure α’ or α modifications are expected to be observed (or the interval where both forms coexist) in this PLA under analysis.

As commented in the Introduction, ternary systems based on PLA as a matrix together with both a plasticizer and a filler, as minor constituents, are not commonly analyzed. Therefore, an exhaustive analysis of the crystallization capability exhibited by these materials is mandatory before the evaluation of any type of properties. Figure 3 shows the DSC curves obtained for the first heating process in all of the examined samples. Several phase transitions are noticeable: glass transition, cold crystallization and melting processes in order of increasing temperatures. It is important to mention that the neat enthalpy involved in the entire DSC curves is negligible in all cases, indicating the absence of crystallinity in the initial extruded samples. That means that these specimens rapidly cooled from the melt show a complete amorphous nature.

**Figure 4:**
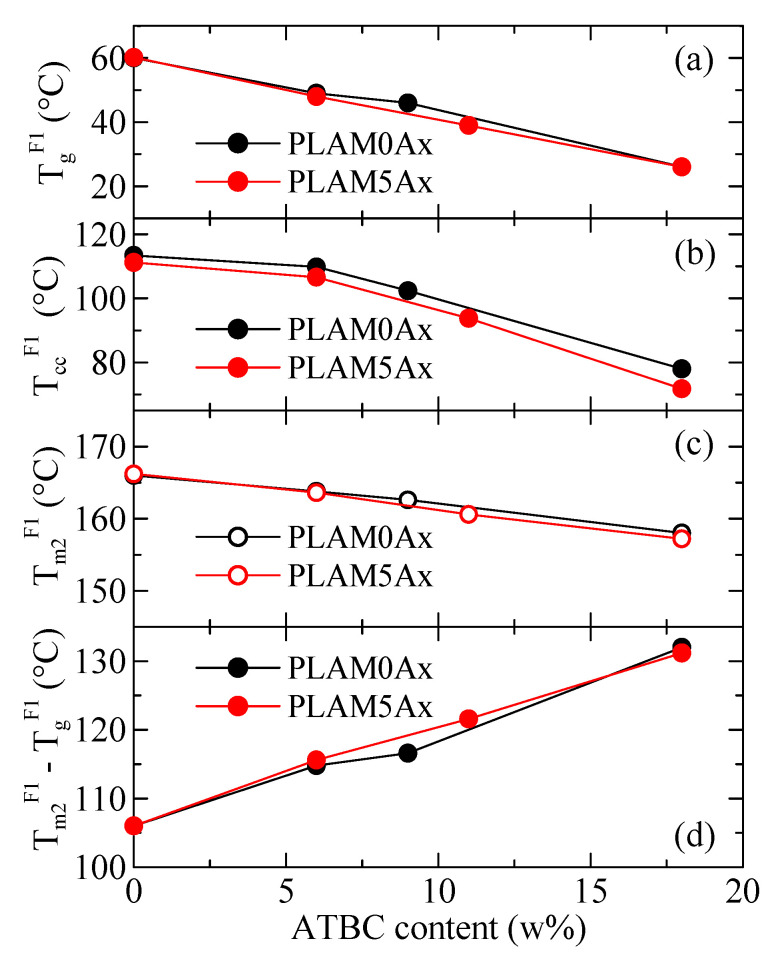
Variation with the ATBC content for the two series of samples of: (**a**) the glass transition temperature; (**b**) the cold crystallization; (**c**) the second melting temperature; (**d**) and the difference between the second melting temperature and the glass transition. All of these magnitudes have been determined in the first heating run of the samples.

Location of the glass transition moves very remarkably to lower temperatures as the ATBC content increases in the samples. In fact, T_g_ depression is around 34 °C, as seen in Figure 3 and quantified in Figure 4a. This reduction is similar to that previously reported in literature [4,28] and it is ascribed to the good compatibility of both PLA and ATBC. The position of T_g_ for the neat ATBC is about −81 °C [28] and, thus, a progressive decrease of the T_g_ in the PLA-ATBC blends is expected as the ATBC content increases if ATBC segregation does not take place.

Furthermore, the different curves show a rather prominent aging peak overlapped at the top of the glass transition. Intensity of this aging peak decreases with increasing ATBC content in the sample, being significantly small for sample PLAM0A20, and negligible for PLAM5A20. One of the reasons for this behavior is that the glass transition of these samples has been moved noticeably to a lower location compared with that for the neat PLA, taking place now at about room temperature (around 25–30 °C). Structural recovery is much faster [29] in these two specimens.

The cold crystallization (T_cc_^F1^) appears at considerably lower temperatures as the ATBC amount rises regarding that shown in the pristine polymeric matrix, meaning that PLA is able to crystallize more rapidly induced by presence of plasticizer. An additional favorable contribution is observed by incorporation of the MCM-41 particles due to their nucleant effect, so that the sample with the smallest T_cc_^F1^ is PLAM5A20 (see Figure 4b). Thus, this nucleating influence is noticed when pure PLA is compared with PLAM5 and when comparison is established between the other plasticized blends with their respective ternary composites, i.e., PLAM0A5 with PLAM5A5 and PLAM0A10 with PLAM5A10.

Reason behind this behavior is believed to be associated with the combined action of incorporation of ATBC and MCM-41 particles within the PLA matrix. On one hand, the MCM-41 silica acts as nucleating agent, similar to the effect observed by addition of mesoporous SBA-15 into PLA [17,18]. This feature allows enhancing crystal nucleation rate due to the reduction of the nucleation energy barrier of PLA via absorption of PLA chains on external and internal surface of MCM-41 [30,31] through the silanol groups existing in this silica [32]. Nevertheless, the crystal growth rate could be constrained by the PLA mobility. Therefore, the T_cc_^F1^ in PLAM5 is expected to decrease due to the addition of MCM-41, as Figure 3 shows. On the other hand, the ATBC improves the mobility of PLA chains by plasticization, as confirmed by the shift of T_g_ values to lower temperatures with increasing ATBC content. Accordingly, the crystal growth rate of PLA should be enhanced when adding MCM-41 and ATBC, turning out in the decrease of the T_cc_^F1^ of PLA, as seen in Figure 3 and Figure 4b.

Moreover, the enthalpy involved in the cold crystallization is also sensitive to the presence of MCM-41 and ATBC. Thus, the values deduced from Figure 3, after normalization to the actual PLA content at each sample, are the following: 38.1, 39.6, 41.9 and 43.8 J/g for the PLA, PLAM0A5, PLAM0A10 and PLAM0A20 specimens, respectively, and 39.2, 40.4, 42.7 and 44.6 J/g for the PLAM5, PLAM5A5, PLAM5A10 and PLAM5A20 materials, respectively. Two conclusions are derived from these values: first, in the two series of samples, PLAM0Ax and PLAM5Ax, the enthalpy increases with the ATBC content, and, second, a further increase is also noted when the MCM-41 is present, so that enthalpies of cold crystallization are in the PLAM5Ax composites higher than those in the PLAM0Ax samples.

On the other hand, according to previous findings [22] reported for this specific PLA, represented in Figure 3 by the vertical lines, the pure α’ modification is expected to be developed in samples PLAM0A20 and PLAM5A20, while the pure α form should be obtained for PLA and PLAM5, with mostly a mixture of the two modifications for the remaining samples. Those findings reported in literature for this specific PLA (with a content in *D*-isomer of about 2 mol%) were based on the dependence on crystallization temperatures of the location for the main reflections derived from X-ray diffraction measurements. The variation in the spacing position of the more intense diffractions was then related to the α′ to α transition [22]. Existence of only the α’ lattice, or of a large amount of this polymorph, can be also characterized by DSC through appearance of the transformation from the α’ to the α crystalline form during the heating process by means of a small exotherm prior the dominant melting process [24,25,33,34]. This transformation is only well noted in PLAM0A20 and PLAM5A20 (at around 130–140 °C), and somewhat in PLAM5A10, as shown in the inset of Figure 3. This observation is in agreement with the statements aforementioned about the expected modification to be obtained.

Figure 3 and Figure 4c show that location of the maximum of melting process also takes place at lower temperatures in these either only plasticized or plasticized and filled materials. The depression in T_m_, of around 9 °C as seen in Figure 4c, is much smaller than the one for T_g_. As a consequence, interval for the actual crystallization occurring at “normal” rates increases considerably on passing from the pristine PLA to PLAM0A20 or PLAM5A20, as noticed in Figure 4d.

**Figure 5:**
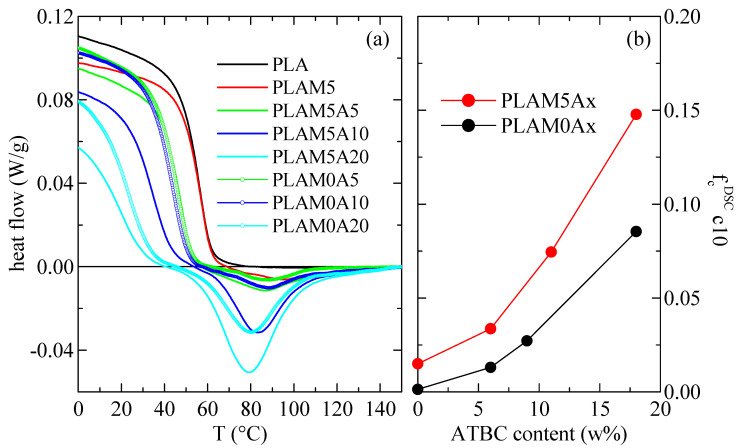
DSC cooling curves (exo down) at 10 °C/min of: (**a**) the different samples and (**b**) DSC crystallinity on cooling at that rate as function of the ATBC content in the sample.

The greater crystallization rate deduced from the results in Figure 3 for the samples plasticized with ATBC and/or filled with MCM-41 is also noted on the cooling curves from the melt represented in Figure 5a. Thus, the neat PLA sample does not show any crystallization at the cooling rate of 10 °C/min, contrary to the other samples, where the exotherm increases in intensity, especially in those materials containing both ATBC and MCM-41. This is also clearly deduced from Figure 5b, where the DSC crystallinity (normalized to the actual PLA content in the material) is represented as function of the ATBC content. A considerable increase in crystallinity is observed for the PLAM0Ax samples, which is even higher for the PLAM5Ax specimens, and, importantly, the difference between the two series is increasingly greater. It is deduced, therefore, that the simultaneous presence of ATBC and MCM-41 leads to an especially noticeable increase of the crystallization rate of PLA. It seems that there is a synergistic effect of mesoporous MCM-41 and ATBC plasticizer on the nucleation ability of PLA, leading not only to a higher crystallization rate, but also extending its crystallization capability.

**Figure 6:**
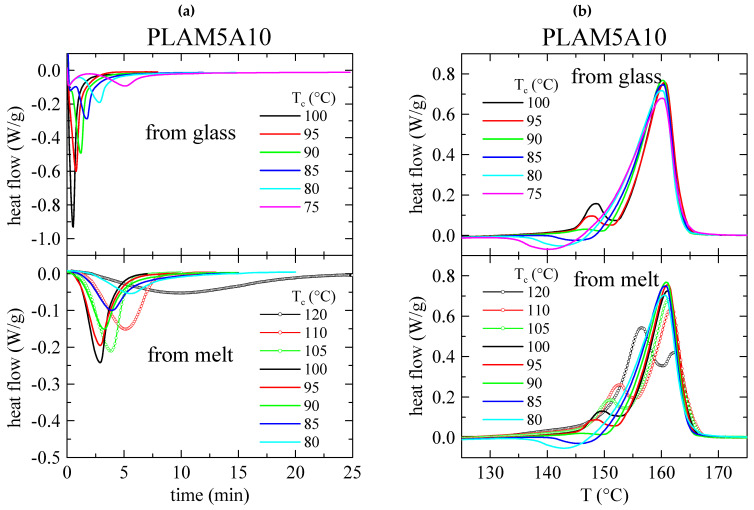
(**a**) Crystallization isotherms (exo down) for sample PLAM5A10, either from the glassy state or from the melt, and (**b**) subsequent heating curves (endo up), at 10 °C/min.

Several experiments of isothermal crystallization have been performed in the different samples by DSC either from the glassy state or from the melt in order to ascertain the increase in crystallization rate. As an example, Figure 6a shows the crystallization isotherms, both from the glassy state or from the melt, and the subsequent heating curves at 10 °C/min (Figure 6b) for sample PLAM5A10.

Important differences are deduced between the isotherms attained from the glass or from melt. Crystallization rate is much faster when it is initiated from the glassy state, as expected, and it increases continuously with the crystallization temperature, in such a way that it is not possible to observe the isotherm above 100 °C, since the crystallization is so fast that it begins during the heating, at 60 °C/min, to reach the isothermal crystallization temperature.

On the contrary, the isotherms from the melt show a crystallization rate considerably much smaller, and the crystallization can be performed up to 120 °C in a reasonable time. Nevertheless, this much lower rate makes the crystallization range in the region of low T_c_s shorter than when crystallization starts from the glass. Moreover, now the crystallization rate shows a maximum at around 100 °C, as expected from the well-known transport and free-energy terms in the crystallization rate [35,36]. A more detailed study of those crystallization rates is made below, extended also to all samples analyzed.

Regarding the subsequent heating, the melting curves for a particular T_c_ are rather similar for both crystallizations, from the glass or from the melt, as observed in Figure 6b. In both cases, the transformation from the α’ to α modification, which is characterized by a melting-recrystallization process [24,25,33,34], is clearly observed until an isothermal T_c_ of around 90 °C. Above that temperature, the melting curves show a bimodal melting endotherm, the low temperature component, termed as T_m1_, supposedly arising from the melting of the real imperfect crystals initially formed at the crystallization temperature, while the second component, T_m2_, is due to the final melting of the recrystallized imperfect crystals [22]. It is interesting to note that the upper limit for observing the transition from the α’ form to the α modification overlaps with the beginning of the observation T_m1_, so that separation between these two processes (the transition α’ to α and T_m1_) at the T_c_ of 90 °C is difficult to be resolved. Again, a more detailed study of these aspects is made below for the different samples.

**Figure 7:**
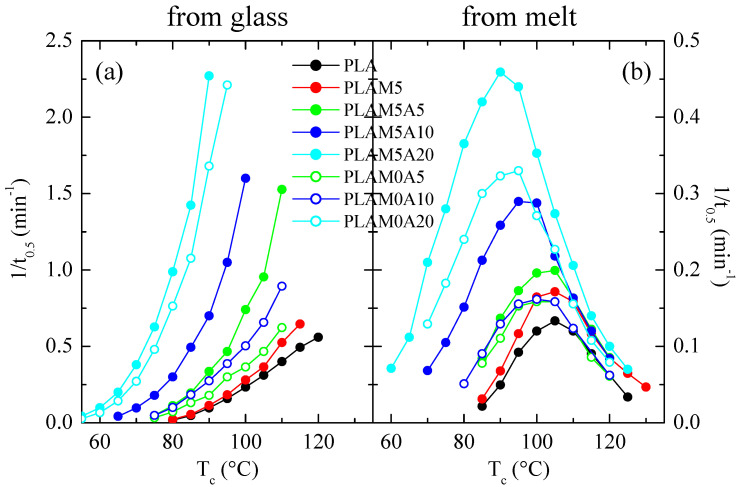
Variation with T_c_ of the inverse of the half crystallization time in the isotherms for the different samples, for isothermal crystallization: (**a**) from the glass and (**b**) from the melt. Note that the Y scale in the data from melt has been amplified 5 times.

Thus, crystallization rate has been estimated from the inverse of time for the 50% transformation in the isotherms, i.e., the half crystallization time, t_0.5_. The values of 1/t_0.5_ are represented in Figure 7 for the different samples and the two modes of crystallization. Crystallization rate is much faster for all the samples when crystallizing from the glass (note that the Y scale in the data from melt has been amplified 5 times in Figure 7b), as mentioned above for PLAM5A10 and as expected. It is observed that, when isothermal crystallization occurs from the glass, the upper limit of T_c_ for reasonable crystallization times occurs at progressively inferior temperatures as the ATBC + MCM41 content increases in the sample (according with the decrease in the cold crystallization temperature obtained in dynamic experiments represented in Figure 3 and Figure 4). And, therefore, the maximum in the crystallization rate is not observed in these specimens crystallized from the glass, since the crystallization is so fast that it begins, as mentioned, during the heating, at 60 °C/min, before reaching the isothermal crystallization temperature.

**Figure 8:**
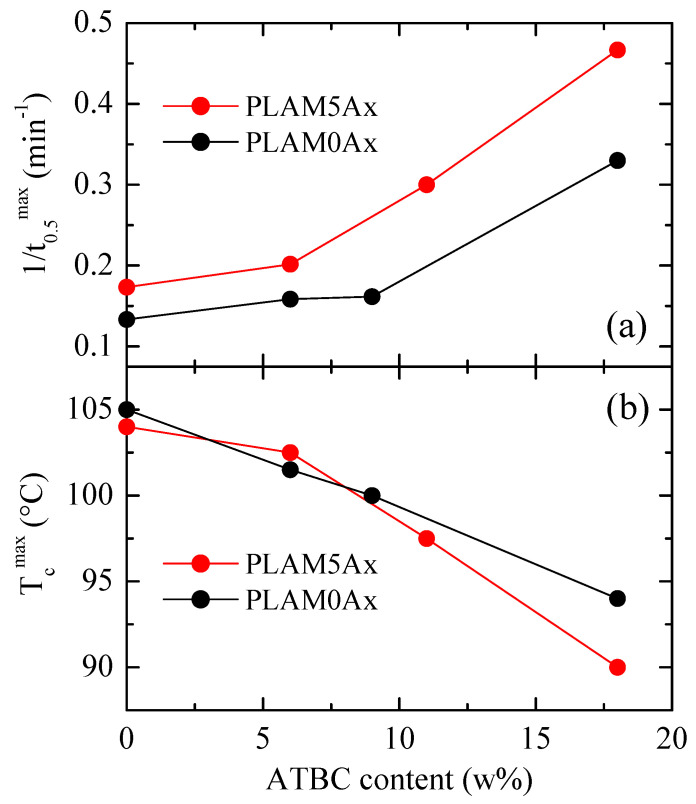
Dependence on the ATBC content in the materials of the maximum rate coordinates in the isotherms from the melt for the two series of samples: (**a**) inverse half crystallization time and (**b**) crystallization temperature at the maximum.

A maximum is, however, noticed for the crystallizations from the melt, as deduced from Figure 7b. This maximum arises from the well-known fact that there are two factors, the free-energy term and the transport one, which determine the crystallization rate of polymers [35,36], as mentioned above. And, these two terms show opposite temperature coefficients. It can be observed that the maximum increases in absolute rate and occurs at progressively lower temperatures as the content in ATBC and MCM-41 is raised. In fact, the coordinates of the maxima, i.e., 1/t_0.5_ and T_c_^max^, are represented in Figure 8 as a function of the ATBC content in the material.

It can be noted that for low contents in ATBC without and with MCM-41, the values of T_c_^max^ are rather similar for the two series, but T_c_^max^ is smaller in the PLAM5Ax specimens at high ATBC amounts. And more importantly, the values for 1/t_0.5_ (the crystallization rate) are significantly higher in the PLAM5Ax series. It follows that there is an important increase in rate when MCM-41 is present, but the increase is much more significant in the composites with both MCM-41 and ATBC. It appears evident again that a synergistic effect of mesoporous MCM-41 silica and plasticizer seems to occur on the nucleation ability of PLA, thus leading to significantly higher crystallization rates. In fact, this rate for PLAM5A20 is analogous to that for efficient nucleants for PLA [37], but now with the advantage that ATBC incorporation decreases significantly the T_g_ of PLA, what has two important implications: first, the crystallization window of PLA is enlarged, and, second, the lower values of T_g_ preclude the physical aging at room temperature, with the consequent influence on the dimensional stability as well as on the expected maintenance of properties with time in these PLA samples [29].

As a final aspect from Figure 7b, there is no clear indication of the possible presence of two different maxima in the crystallization rate: one for the disordered α’ modification and another for the ordered α form.

**Figure 9:**
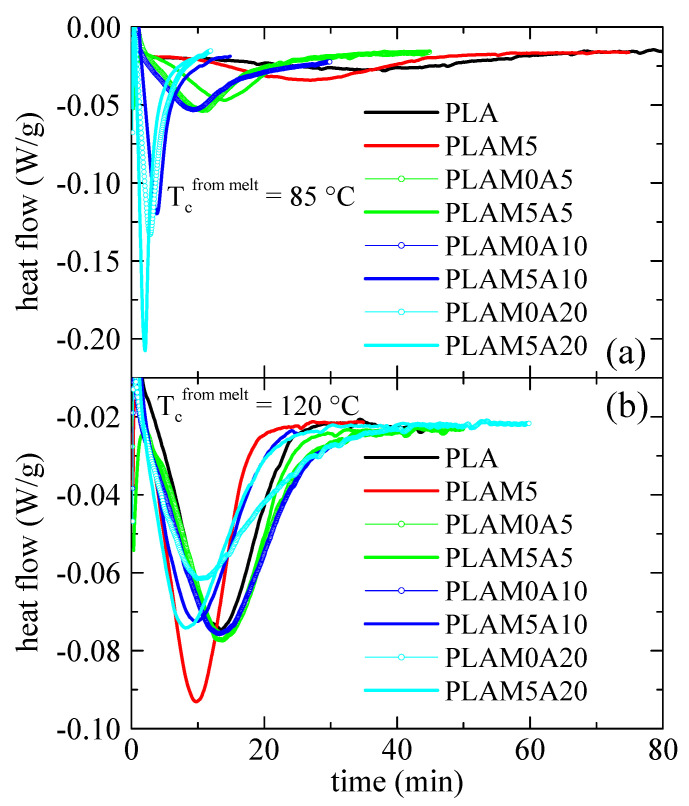
Crystallization isotherms (exo down) from the melt for the different samples at (**a**) T_c_ = 85 °C, and (**b**) 120 °C.

**Figure 10:**
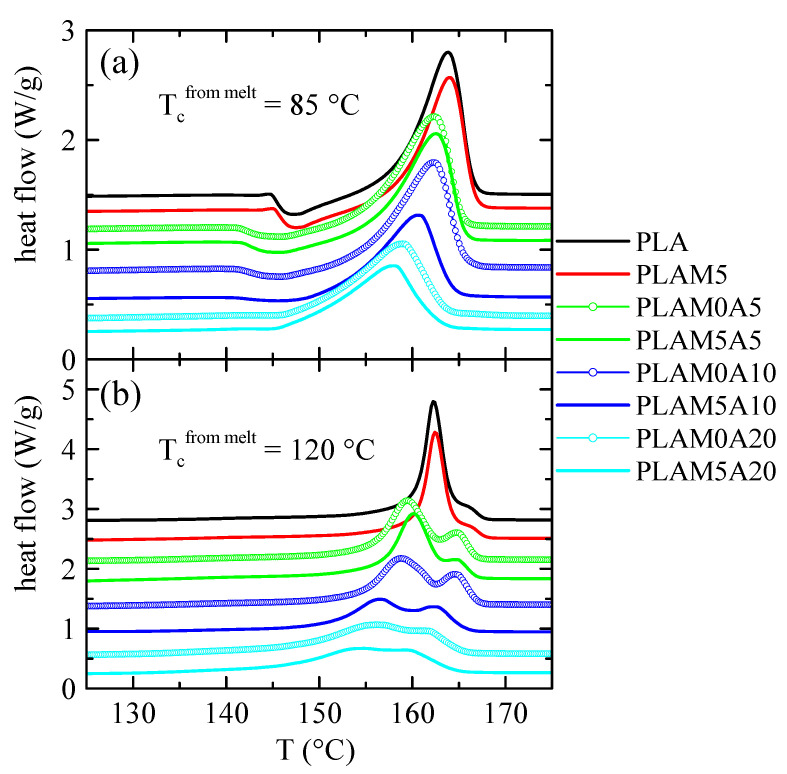
DSC melting curves (endo up) at 10 °C/min for the different samples after crystallization from the melt at (**a**) T_c_ = 85 °C, and (**b**) 120 °C.

In order to compare the behavior of the different materials, two values of T_c_ have been chosen for the samples isothermally crystallized from the melt where there are experimental values for all of the samples. The lower isothermal T_c_ selected corresponds with one that allows formation of the neat disordered α’ modification, while the more ordered α form is developed at the other T_c_, the highest one. These temperatures are 85 and 120 °C, respectively, and the results for the crystallization isotherms and for the subsequent heating ramps are shown in Figure 9 and Figure 10, respectively. Regarding the isotherms, Figure 9a indicates that for T_c_ = 85 °C there is a very important increase of the crystallization rate in materials containing MCM-41 with increasing ATBC amounts. The differences among the samples are, however, much smaller in the case of T_c_ = 120 °C (Figure 9b). Moreover, it is noteworthy that the isotherm for sample PLAM5 is considerably narrower than the one for PLA.

The subsequent melting curves show rather interesting features. Concerning the curves of materials isothermally crystallized at T_c_ = 85 °C (Figure 10a), the α’ to α transition occurs for samples PLA and PLAM5 at higher temperatures than for the other specimens. Moreover, this transformation is more clearly noticeable (the apparent heat flow change is higher) in those two samples, while the heat flow change is progressively decreasing in such a way that is rather small (but appreciable) for sample PLAM5A20. It is true, however, that the DSC curves in Figure 10 are the actual curves, not normalized to the real PLA content in the sample. Furthermore, there is continuous decrease of the main peak temperature on passing from PLA to PLAM5A20 (see below the corresponding values), in the melting endotherms.

Regarding the results for T_c_ = 120 °C, several features are deduced from Figure 10b. Firstly, the two constituents of the now bimodal melting endotherms also decrease in temperature from PLA to PLAM5A20 (see below). Secondly, the recrystallized component (T_m2_) is increasing considerably its intensity in relation to T_m1_ as the content in ATBC increases and MCM-41 is added. And, finally, the two melting peaks are getting wider as that content is raised. It seems, therefore, that the presence of ATBC leads to a wider distribution in the size of the crystallites.

**Figure 11:**
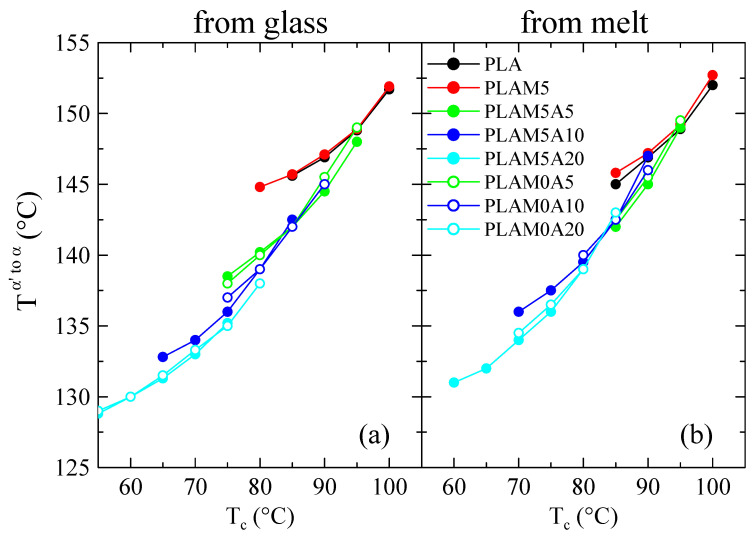
Variation with T_c_ of the temperature for transition from the α’ to α for the different samples, when crystallizing (**a**) from the glass, and (**b**) from the melt.

**Figure 12:**
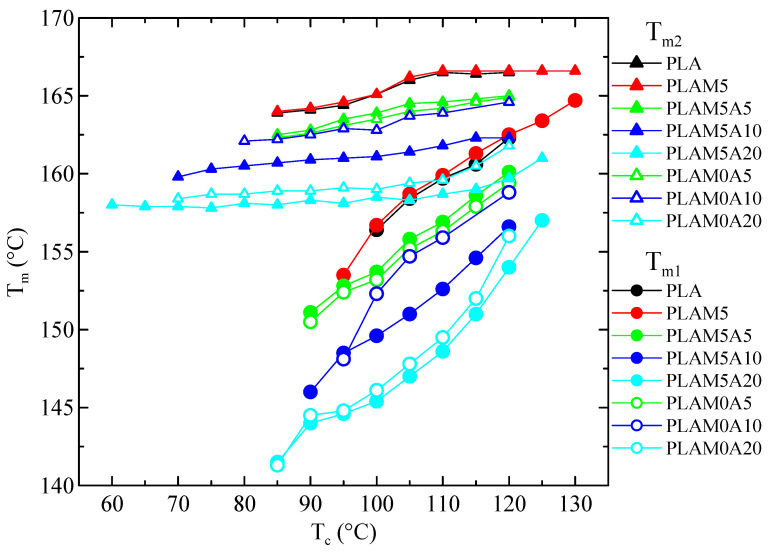
Variation with T_c_ of the melting temperatures for the different samples, when crystallizing from the melt: T_m1_ (circles) and T_m2_ (triangles).

The results for all the materials and crystallization temperatures regarding the α’ to α transition temperature together with the melting temperatures are represented in Figure 11 and Figure 12, respectively. The temperatures for the transition from the α’ to α modification, displayed in Figure 11, indicate, first, that rather similar values are obtained when crystallizing from the glass or from the melt. Moreover, a small, but appreciable, decrease of those temperatures is observed as the ATBC content increases, extending also continuously the interval of actual crystallization temperatures.

Regarding the melting temperatures (Figure 12), the values of T_m1_ for the low T_c_’s appear to decrease abnormally, since they are overlapped to the transition from the α’ form to the α polymorph, as commented. For higher T_c_’s, however, a continuous increase in the values of T_m1_ is observed for all materials, in such a way that they tend to those of T_m2_, where the variation with T_c_ is much smaller. Moreover, and comparing the different samples, the presence of ATBC and MCM-41 leads to significantly smaller values for the two melting temperatures (although this decrease is not as high as the one for T_g_, as seen in Figure 4).

**Figure 13:**
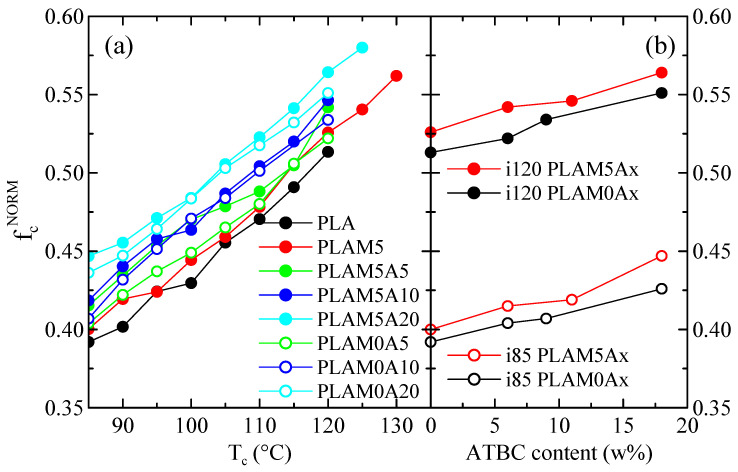
Variation of the DSC crystallinity, normalized to the actual PLA content, for the experiments conducted from the melt, as a function of T_c_ for the different samples (**a**), and of the ATBC content after isothermal crystallization at 85 and 120 °C for the two series of samples (**b**).

The values of the PLA crystallinity developed in the different materials and crystallization conditions have been estimated from the enthalpies involved in the corresponding melting curves and after normalization to the actual PLA content in the sample. For instance, Figure 13a displays the values of the so determined DSC crystallinity as a function of the crystallization temperature in the experiments conducted from the melt. It can be observed that there is a more or less continuous increase of the crystallinity with the ATBC + MCM content in the sample. A more clear picture is deduced from Figure 13b, representing the variation with the ATBC content of the degrees of crystallinity after isothermal crystallization at 85 and 120 °C for the two series of samples. It is evident, firstly, that the presence of ATBC leads to an increase of the crystallinity of around 0.02–0.03 units at the maximum ATBC content compared with the neat PLA. Moreover, there is an additional increase of 0.01–0.02 units more when mesoporous MCM-41 particles are also present. Again, the simultaneous presence of ATBC and MCM-41 involves a beneficial effect, now in the crystallinity values, in these materials.

**Figure 14:**
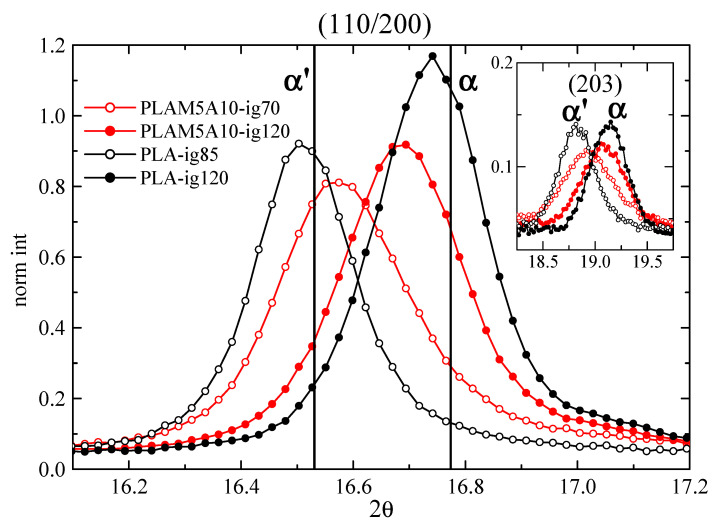
Room-temperature X-ray diffractograms of different samples after cold crystallization from the amorphous glassy state at the indicated temperatures, showing the angular interval of the main (110/200) diffraction and that for the (203) reflection one in the inset. The vertical lines are the corresponding values reported in ref. [33] for the pure α’ and α modifications.

As a final aspect, some preliminary X-ray diffraction experiments have been performed in samples isothermally crystallized in the DSC and brought to room temperature at the end of the isotherm. Two representative materials (neat PLA and PLAM5A10) have been crystallized from the glass at two temperatures, and the corresponding X-ray diffractograms are shown in Figure 14. It is observed that the diffraction for the sample of PLA cold crystallized at 85 °C leads to a (110/200) diffraction peak centered very close to that reported for the pure α’ modification [33], while this reflection in the specimen isothermally crystallized at 120 °C is close to the characteristic for the α modification. Similar features apply for the (203) diffraction, shown in the inset of Figure 14. Concerning the PLAM5A10 material cold crystallized at 70 and 120 °C, these profiles are close to the α’ and α forms, respectively, but not as close as the PLA samples. Moreover, the PLAM5A10 profiles display a somewhat higher width. The reason for this behavior may be related to the fact that the DSC pans for performing these X-ray measurements were not hermetically close in order to facilitate the specimens removing prior X-ray experiments. That may lead to some uncertainty in the real temperatures inside the sample. Experiments are being planned to study the crystallization of the different samples under real-time variable-temperature X-ray conditions using synchrotron radiation, and thus analyze the possible differences of the crystalline modifications between PLA and the materials with ATBC and MCM-41. Furthermore, a complete evaluation of several mechanical properties is under progress in order to analyze and understand the influence of presence of the selected plasticizer and nucleant agent in the final performance of these, either fully amorphous or isothermally crystallized, plasticized and/or filled materials.

## 4. Conclusions

Binary blends and dual compounds, the former based on an *L*-rich PLA and ATBC as plasticizer and the latter on PLA and MCM-41 as nucleating agent, together with ternary composites consisting of PLA, ATBC at several compositions and MCM-41 silica at a given content, were processed by melt extrusion. An appropriate distribution of mesoporous particles was found in the different loaded materials along with the absence of bulky silica domains.

The combined effect of ATBC and MCM-41 silica leads to an increase of the PLA thermal stability, showing the optimized situation for the sample PLAM5A10

Several phase transitions are noticeable by DSC during the first heating process under dynamic conditions in all of the materials: glass transition, cold crystallization and melting processes in order of increasing temperatures. Location of the glass transition moves very remarkably to lower temperatures as the ATBC content increases in the samples. Motion capability of amorphous phase is not, however, affected at a given plasticizer amount by the incorporation of MCM-41 in the ternary systems.

The cold crystallization is also considerably shifted to lower temperatures as the ATBC amount rises, in relation to that shown in the pristine matrix. Thus, PLA is able to crystallize more rapidly boosted by presence of plasticizer. Furthermore, an additional favorable contribution is provided by inclusion of the MCM-41 particles in the ternary composites. Position of the maximum of melting process also takes place at lower temperatures in these either only plasticized or plasticized and loaded systems, this decrease being, however, much smaller than the one for T_g_. As a consequence, the crystallization window is considerably enlarged by the presence of ATBC and MCM-41.

A higher crystallization rate is deduced by addition of the plasticizer and/or mesoporous silica under dynamic cooling at 10 °C/min. The neat PLA does not show any crystallization at that cooling rate, contrary to the other materials. Thus, a considerable increase in crystallinity is observed for the PLAM0Ax samples, which is even higher for the PLAM5Ax specimens.

PLA crystallization rate in isothermal experiments is much faster from the glassy state than from the melt, as expected. A maximum rate is found at around 90–105 °C in the experiments conducted from the melt, showing a very important rise of the crystallization rate as the content in ATBC and MCM-41 increases. That maximum in the rate is, however, not observed in the specimens crystallized from the glassy state, since the crystallization is so fast that it begins during the heating before reaching the isothermal crystallization temperature.

Summarizing, a synergistic effect of mesoporous MCM-41 silica and ATBC plasticizer seems to occur on the nucleation ability of PLA, thus leading to considerably higher crystallization rates and broadening the crystallization window of this PLA.

## Figures and Tables

**Table 1:** Specimen labels and nominal composition for the different materials under study.

Sample	Nominal Material Composition (wt.%)		
	PLA	MCM-41	ATBC
PLA	100	0	0
PLAM0A5	95	0	5
PLAM0A10	90	0	10
PLAM0A20	80	0	20
PLAM5	95	5	0
PLAM5A5	90	5	5
PLAM5A10	85	5	10
PLAM5A20	75	5	20

**Table 2:** Results derived from TGA experiments performed under air: temperature for 1% degradation (T_1%_), the maximum degradation temperature (T_max_^PLA^) and the actual material content based on TGA analysis for the different constituents: PLA, ATBC plasticizer and MCM-41 silica.

Specimen	T_1%_ (°C)	T_max_^PLA^ (°C)	TGA Content (wt.%)		
			PLA	MCM-41	ATBC
PLA	221.0	315.5	100	0	0
PLAM0A5	159.0	338.0	94	0	6
PLAM0A10	154.0	341.5	91	0	9
PLAM0A20	148.0	341.5	82	0	18
PLAM5	260.0	358.5	95.3	4.7	0
PLAM5A5	161.0	337.0	90.1	3.9	6
PLAM5A10	149.0	358.0	83.7	5.3	11
PLAM5A20	145.0	344.0	77.2	4.8	18

## Data Availability

Data are contained within the article.
